# Caffeine supplementation improves physical performance without affecting fatigue level: a double-blind crossover study

**DOI:** 10.5114/biolsport.2022.107479

**Published:** 2021-07-08

**Authors:** Yuri Campos, Ángel Lago-Rodríguez, Alejandro F. San Juan, Víctor Moreno-Pérez, Alvaro Lopez-Samanes, Antonio J. Sánchez-Oliver, Sandro F. Da Silva, Raúl Domínguez

**Affiliations:** 1 Postgraduate Program of the Faculty of Physical Education and Sports of the University of Juiz de Fora, Brazil; 2 Study Group and Research in Neuromuscular Responses, University of Lavras, Brazil; 3 Faculty of Health Sciences, University Isabel I, Spain; 4 Department of Health and Human Performance, Sport Biomechanics Laboratory, Faculty of Physical Activity and Sports Sciences-INEF, Universidad Politécnica de Madrid, Spain; 5 Center for Translational Research in Physiotherapy, Department of Pathology and Surgery, Miguel Hernández University; 6 School of Physiotherapy, Faculty of Health Sciences, Universidad Francisco de Vitoria, Spain; 7 Departamento de Motricidad Humana y Rendimiento Deportivo, Universidad de Sevilla, Spain

**Keywords:** Countermovement jump, Ergogenic aids, Glycolytic metabolism, Lactate, Sport nutrition, Wingate

## Abstract

This study examined the effect of caffeine supplementation (CAFF) in a Wingate test (WT), and the behaviour of blood lactate concentrations (BLa) and neuromuscular fatigue (NMF), measured as reduced countermovement jump (CMJ) performance, in response to the WT. In a double-blind crossover study, 16 participants attended the laboratory twice, separated by a 72-hour window. In the sessions, participants first ingested 6 mg·kg^-1^ of either CAFF or placebo (PLAC), and then performed a WT. BLa was measured before (L-pre), and 0.5 min (L-post-0.5) and 3.5 min (L-post-3.5) after conducting the WT. The CMJ test was conducted before (CMJ pre), after (CMJ post), and 3 min after completing (CMJ post-3) the WT. The results indicated that CAFF enhanced peak power (Wpeak: + 3.22%; *p* = 0.040), time taken to reach Wpeak (T_Wpeak: -18.76%; *p* = 0.001) and mean power (Wmean: + 2.7%; *p* = 0.020). A higher BLa was recorded for CAFF at L-post-0.5 (+ 13.29%; *p* = 0.009) and L-post-3.5 (+ 10.51%; *p* = 0.044) compared to PLAC. CAFF improved peak power (PP; + 3.44%; *p* = 0.003) and mean power (MP; + 4.78%; *p* = 0.006) at CMJ pre, compared to PLAC, whereas PP and MP were significantly diminished at CMJ post and CMJ post-3 compared to pre (*p* < 0.001 for all comparisons) under both the CAFF and PLAC conditions. PP and MP were increased at post-3 compared to post (*p* < 0.001 for all comparisons) for both conditions. In conclusion, CAFF increased WT performance and BLa without affecting NMF measured by CMJ. Thus, CAFF may allow athletes to train with higher workloads and enhance the supercompensation effects after an adequate recovery period.

## INTRODUCTION

Caffeine (CAFF) is one of the most widely used sport supplements. An example is that some 97% of professional soccer clubs in the UK provide their players with caffeine to improve performance [1]. In the 1970s, the first studies that analysed the effect of CAFF on sports performance reported an improvement in time to exhaustion (TTE), attributing this ergogenic effect to increased lipolysis and sparing of muscle glycogen. In this regard, in vitro studies have observed that CAFF leads to high calcium (Ca^2 +)^ bioavailability in the myoplasm through an effect on the velocity of the rate of release from the sarcoplasmic reticulum [2]. However, CAFF’s ergogenic effect is currently mainly attributed to its antagonistic function to adenosine receptors, as CAFF has a high binding capacity to these receptors. Thus, through the blockade of receptors A_1_, A_2a_, and A_2b_ [3], CAFF prevents the inhibitory effects of adenosine on neuro-excitability, increasing the synthesis of excitatory neurotransmitters in the brain (particularly dopamine), and promoting analgesic effects that could help to reduce the rate of perceived exertion (RPE) [4], along with improved cognitive function [5]. Furthermore, although there are reports that CAFF supplementation improves performance in endurance sports with a predominance of aerobic metabolism [6], interest in the possible ergogenic effects of CAFF on anaerobic exercise efforts has increased.

When executing high-intensity anaerobic exercises, concentrations of blood lactate (BLa) and hydrogen ions (H ^+)^ rise, lowering the pH of the muscle cell [7] and reducing its capacity to generate adenosine triphosphate (ATP) for muscle contraction [8]. One of the most valid and reliable tools used to assess anaerobic performance is the 30-s Wingate test (WT). This test assesses the capacity of the muscle to generate power through anaerobic energy systems [9]. The accumulation of H ^+^ during WT inhibits phosphocreatine (PCr) resynthesis and phosphofructokinase activity responsible for the main stages of glycolytic metabolism leading to muscle fatigue [10]. In this way, recent studies have also highlighted that CAFF can improve mean and peak power registered during the WT [11].

The decline in the ability to generate strength and/or power output with repetitive stimuli, measured through an objective performance marker, can be defined as fatigue [12]. The countermovement jump (CMJ) test is commonly used in high-performance sport athletes in order to monitor neuromuscular fatigue (NMF) [13], recovery status [14], and training-induced adaptations [15]. The CMJ test can be used to repeatedly assess jump ability over a short period of time in an individual, promoting scarce physiological strain, which makes it a very useful tool to measure muscular fatigue [16]. Power and force produced during CMJ, along with jump height – which is directly associated with jump total time – are indicators of lower limb muscular power output [17]. Diminished post-exercise CMJ performance is considered appropriate to monitor neuromuscular status [18]. In addition, the incorporation of metabolic variables, such as BLa concentration, helps to improve our understanding of the post-exercise fatigue recovery process [14].

For athletes practising anaerobic sport modalities, it needs to be stablished whether CAFF can be considered ergogenic during competition and training as any possible ergogenic effect will likely affect post-exercise NMF. Accordingly, the aims of the present study were: (a) to examine the ergogenic effect of CAFF on WT performance and (b) to determine its impacts on post-exercise BLA, and fatigue measured thorough loss of CMJ ability. The hypothesis of our study was that CAFF would increase performance in a WT and BLa concentration without affecting post-exercise NMF.

## MATERIALS AND METHODS

### Design of the study

**FIG. 1:**
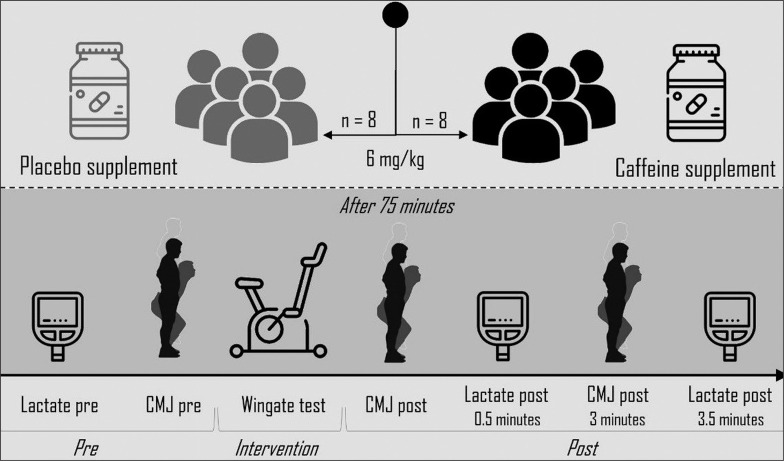
Summary of the experimental session. CMJ: countermovement jump.

In a double-blind crossover study, participants attended twice in a 72-hour window, within the same time frame (± 0.5 hours) to avoid bias due to the circadian rhythm interaction associated with caffeine intake [19]. In each session, 50% of the participants were randomly assigned to CAFF or placebo (PLAC) supplementation (www.randomizer.org). Each experimental session consisted of a WT, and measurement of BLa and CMJ performance to assess metabolic and neuromuscular recovery, respectively. Thus, BLa concentrations was measured before (L-pre), 0.5 (L-post), and 3.5 (L-post-3.5) minutes after completing the WT. Jump ability was assessed before and after the WT (CMJ post) and after 3 min after the WT test (CMJ post-3) (see Figure 1).

### Participants

Sixteen male degree sports science students participated in the study (age: 22.69 ± 2.12 years; height: 1.78 ± 0.06 m; weight: 78.09 ± 10.27 kg; body mass index: 24.72 ± 2.74 kg·m^-2^). In order to participate in the study, researchers checked in an informative session that all the volunteers fulfilled the following inclusion criteria: i) continuous resistance training with a minimum of 3 sessions per week during at least 18 months before the study; ii) resistance training performance: their one-repetition maximum strength (1RM) normalized per kg of body mass was 1 for bench press and >1.5 for the back squat exercise; iii) no sports supplements taken in the 3 months leading up to the study outset; iv) not smoking; v) not considered to be an elite athlete; vi) not being diagnosed with a cardiovascular, respiratory, or metabolic disease; vii) no orthopaedic problem that could affect cycling technique; viii) previous experience with performing the WT. All subjects were informed of the procedure and the nutritional guidelines before giving their written informed consent. The study protocol fulfilled ethical international standards as approved by the Ethics Committee of the University Alfonso X El Sabio (code number 1.010.704).

### Supplementations and dietary control

Participants arrived at the laboratory 75 min before starting WT and were instructed to take the corresponding CAFF (6 mg·kg^-1^) or PLAC (6 mg·kg^-1^ of sucrose) supplement. Capsules used were no. 1 opaque red (Guinama S.L.U, 0044634, La Pobla de Valbona, Spain) with a capacity of 0.50 mL/capsule where a researcher adjusted the individualized content of CAFF or PLAC using a semi-automatic manual filling machine Capsunorm 2000 (Miranda de Ebro, Spain). Supplementation timing was based on the time needed to achieve peak plasma caffeine levels (60 minutes) [20] and values reported on a disaggregation quality assay (13.4 min) [21]. To ensure similar nutritional intake (60% carbohydrates, 30% lipids, and 10% proteins) from 72 hours before the study onset, until its end, participants were provided with a set of guidelines. Furthermore, since the half-life for caffeine elimination has been reported in the range of 2.5–10 hours [22], caffeine intake was restricted 24 h before each experimental session in order to avoid potential interaction with the study results. Hence, participants were given a list of foodstuffs rich in caffeine (coffee, tea, mate, energizing drinks, cola drinks, chocolate drinks, and chocolate) that should be avoided.

### Wingate test

The WT was executed on a Monark cycle ergometer (Ergomedic 828E, Vansbro, Sweden) and it was preceded by a standardized warm-up. The WT procedure has been described elsewhere [14]. During the test, power values (W) were recorded every second. For data analysis, peak power (W_peak_), time (s) taken to reach W_peak_ (T_W_peak_), mean power (W_mean_), and minimum power (W_min_), considered as the minimum W registered in the last 10 s of the test, were extracted. Mean power was also calculated for 6 five-second splits of 5 seconds during the WT (W_split_0–5_, W_split_5–10_, W_split_10–15_, W_split_15–20_, W_split_20–25_, and W_split_25–30_).

### Blood lactate

Before the warm-up (L-pre), and at 0.5 min (L-post-0.5) and 3.5 min (L-post-3.5) after completing the WT, an evaluator extracted a blood sample (5 μl) from the tip of the index finger of the left hand for BLa determination using a Pro 2 LT-1710 analyser (Arkray Factory Inc., KDK Corporation, Shiga, Japan).

### Neuromuscular fatigue

NMF changes were determined through performance of a CMJ measured before and after the WT following a previously described method [23]. Using a force platform (Quattro Jump model 9290AD; Kistler Instruments, Winterthur, Switzerland), each participant performed two CMJs, separated by a 45 s rest period, at three time points: before (CMJ pre) the WT, just after finishing the WT (CMJ post), and after 3 min later (CMJ post-3). These variables recorded in this test were average values of the two CMJs for jump total time (TT), mean power (MP) and peak power (PP).

### Statistical analysis

Data are presented as mean ± standard deviation (SD). A *t*-test for related samples was performed for normally distributed variables (tested with the Kolmogorov-Smirnov test) and the Wilcoxon test for those showing a non-normal distribution (as confirmed by the Kolmogorov-Smirnov test) in the WT. A Supplementation by Time analysis of variance for repeated measures (ANOVA-RM) was applied for BLa and NMF, whereas a Supplementation by Split ANOVA-RM was applied for split-specific average power. Greenhouse-Geiser corrections were applied for non-spherical distributions, evaluated using Mauchly’s test, and Bonferroni corrections were applied for post-hoc comparisons. Cohen’s d effect size was calculated for pairwise comparisons, with values > 0.8, 0.5–0.8, 0.2–0.5 and < 0.2 considered as large, moderate, small, and trivial, respectively [24]. Partial eta squared ($\eta_{p}^{2}$) was calculated for ANOVA-RM where < 0.25, 0.26–0.63 and > 0.63 were considered small, medium and large effect sizes, respectively [25]. Tests were performed using IBM SPSS Statistics for Mac, version 20.0 (IBM Corp., Armonk, NY, USA). Significance was set at *p* ≤ 0.05.

## RESULTS

### Wingate test performance

Increased W_peak_ (*p* = 0.04; *d* = 0.56) and W_mean_ (*p* = 0.02; *d* = 0.64), along with reduced T_W_peak_ (*p* = 0.001; *d* = 1.08), were observed in the WT for the CAFF condition, compared to PLAC. No differences were observed in W_min_ (*p* = 0.76) (see Table 1).

**TABLE 1:** Effects of CAFF supplementation on power output during WT. Values are represented as mean ± standard deviation [95% confidence interval].

	PLAC		CAFF		Δ%	t	*p*	d
	M + SD [95% CI]	CV (%)	M + SD [95 % CI]	CV (%)				
**W_peak_ (W)**	885.88 ± 155.1 [803.23, 968.52]	17.5	914.38 ± 153.16 [832.76, 995.99]	16.8	+ 3.22	-2.241	0.040*	0.56
**T_W_peak_ (s)**	8.69 ± 1.45 [7.92, 9.46]	16.7	7.06 ± 1.29 [6.38, 7.75]	18.3	-18.76	4.333	0.001*	1.08
**W_mean_ (W)**	677.05 ± 106.12 [620.51, 733.6]	15.7	695.33 ± 110.74 [636.32, 754.34]	15.9	+ 2.7	-2.568	0.020*	0.64
**W_min_ (W)**	477 ± 77.53 [435.69, 518.31]	16.3	471.56 ± 99 [418.81, 524.31]	21.0	-1.14	0.312	0.760	0.08

Note: W_peak_: peak power; T_W_peak_: time to reach W_peak_; W_mean_: mean power; W_min_: minimum power; M: mean; SD: standard deviation; 95% CI: 95% confidence interval; CV (%): coefficient of variation; Δ%: percentage difference from PLAC; *d*: Cohen’s *d* effect size.

An effect of Supplementation (F_1,15_ = 12.19; *p* = 0.003; $\eta_{p}^{2}$ = 0.45) and Time (F_1.7, 25.6_ = 101.21; *p* < 0.001; $\eta_{p}^{2}$ = 0.87), with no Supplementation by Time interaction (F_1.2, 17.7_ = 2.7; *p* = 0.113; $\eta_{p}^{2}$ = 0.15), was found for mean power recorded in 6 splits of 5 seconds each during the WT. Overall, greater mean power output was observed for CAFF, compared to PLAC (725.73 ± 130.16 vs. 697.54 ± 131.6 W). Table 2 details the results of comparing average data among splits.

**TABLE 2:** Mean power output registered in splits of 5 s during Wingate test. Values are represented as mean ± standard deviation [95% confidence interval].

	PLAC		CAFF		Statistical significance (p-value)		
	M ± DS [CI 95%]	CV (%)	M ± DS [CI 95%]	CV (%)	S	T	S × T
**split_0–5_**	585.18 ± 124.20 [519, 651.36]	21.22	625.35 ± 124.37 [559.08, 691.62]	19.89			
**split_5–10_ ^#^**	724.95 ± 135.33 [652.83, 797.06]	18.67	760.33 ± 131.95 [690.02, 830.64]	17.35			
**split_10–15_^#π^**	750.20 ± 130.58 [680.62, 819.78]	17.41	779.61 ± 127.23 [711.82, 847.41]	16.32	0.003	< 0.001	0.113
**split_15–20_^#γ^**	737.82 ± 123.05 [672.25, 803.39]	16.68	762.44 ± 122.17 [697.34, 827.54]	16.02			
**split_20–25_^#γ δ^**	710.03 ± 113.69 [649.45, 770.62]	16.01	731.32 ± 116.00 [669.5, 793.13]	15.86			
**split_25–30_^#π γ δ τ^**	677.05 ± 106.12 [620.51, 733.6]	15.67	695.33 ± 110.74 [636.32, 754.34]	15.93			

Note: PLAC: placebo; CAFF: caffeine; S: supplementation; T: time; M: mean; SD: standard deviation; 95% CI: 95% confidence interval; CV (%): coefficient of variation;

# : significantly different (*p* < 0.001) from split_0–5_;

π : significantly different (*p* < 0.001) from split_5–10_;

γ : significantly different (*p* < 0.001) from split_10–15_;

δ : significantly different (*p* < 0.001) from split_15–20_;

τ : significantly different (*p* < 0.001) from split_20–25_.

### Blood lactate concentration

**FIG. 2:**
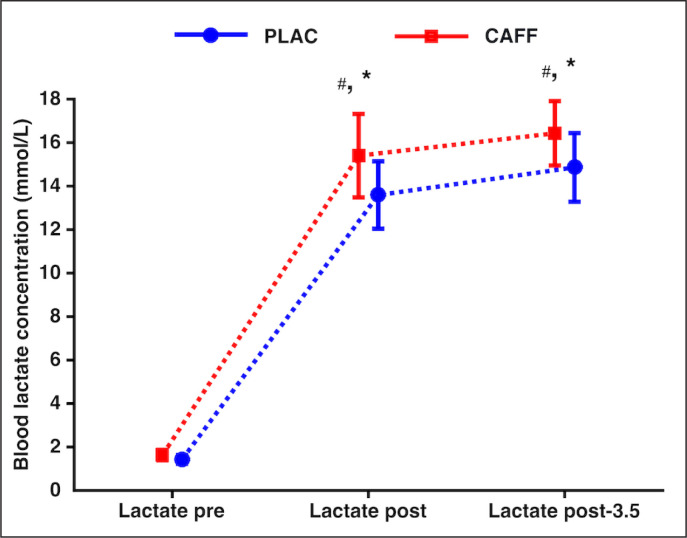
Mean ± 95% confidence intervals for blood lactate concentration registered at Lactate pre, Lactate post-0.5, and Lactate post-3.5 are shown in this figure. *: significant differences from Lactate pre (*p* < 0.05); #: significant differences between CAFF and PLAC (*p* < 0.05).

Our analysis revealed a Supplementation by Time interaction (F_2,30_ = 3.837; *p* = 0.033; $\eta_{p}^{2}$ = 0.204), and an effect of Supplementation (F_1,15_ = 8.621; *p* = 0.01; $\eta_{p}^{2}$ = 0.365) and Time (F_2,30_ = 291.912; *p* < 0.001; $\eta_{p}^{2}$ = 0.951). BLa was found to be increased (*p* < 0.001) at L-post-0.5 and L-post-3.5, under both the CAFF and PLAC conditions. In addition, BLa was greater in the CAFF condition at L-post-0.5 (Δ% = 13.29; *p* = 0.009) and L-post-3.5 (Δ% = 10.51; *p* = 0.044), vs. PLAC (see Figure 2).

### Neuromuscular fatigue

A Time effect was observed for CMJ TT (F_2,30_ = 3.380; *p* = 0.047; $\eta_{p}^{2}$ = 1.84), with larger CMJ post values recorded compared to CMJ post-3 (0.80 ± 0.14 vs. 0.75 ± 0.11; *p* = 0.049). No Supplementation by Time interaction (F_2,30_ = 0.034; *p* = 0.967; $\eta_{p}^{2}$ = 0.002) or Supplementation effect (F_1,15_ = 0.116; *p* = 0.739; $\eta_{p}^{2}$ = 0.008) was observed.

**FIG. 3:**
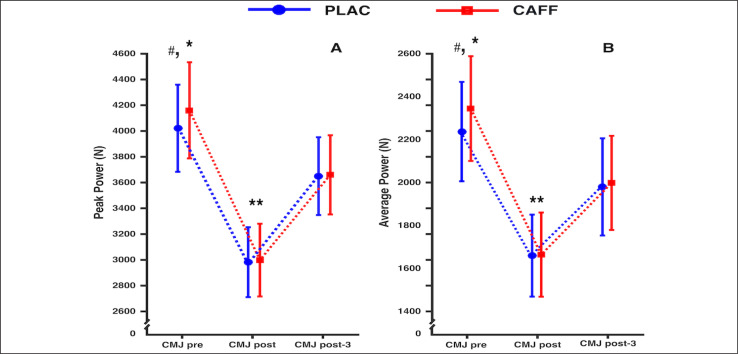
Mean ± 95% confidence intervals for PP (A) and MP (B) registered at CMJ pre, CMJ post, and CMJ post-3 are shown in this figure. *: significant difference (*p* < 0.001) compared to post and post-3 for CAFF and PLAC; **: significant difference (*p* < 0.001) compared to post-3 for CAFF and PLAC; #: significant difference (*p* < 0.01) between CAFF and PLAC.

A Supplementation by Time interaction (F_2,30_ = 3.318; *p* = 0.05; $\eta_{p}^{2}$ = 0.181) and Time effect (F_1.298,19.385_ = 130.209; *p* < 0.001; $\eta_{p}^{2}$ = 0.897) were found for PP, with no Supplementation effect (F_1,15_ = 3.531; *p* = 0.08; $\eta_{p}^{2}$ = 0.191) (Figure 3A). For both conditions, CAFF and PLAC, greater values (*p* < 0.001) were found at the time point CMJ pre, compared to CMJ post and CMJ post-3, and smaller values (*p* < 0.001) were noted at CMJ post, compared to CMJ post-3. Moreover, a higher PP was recorded for CAFF at CMJ pre, compared to PLAC (4160.21 ± 700.23 W vs. 4021.79 ± 634.29 W; p = 0.003; Figure 3A).

A Supplementation by Time interaction (F_2,30_ = 3.975; *p* = 0.029; $\eta_{p}^{2}$ = 0.209), and Time effect (F_1.391, 20.862_ = 104.279; *p* < 0.001; $\eta_{p}^{2}$ = 0.874) were observed for MP, with no Supplementation effect (F_1,15_ = 3.458; *p* = 0.083; $\eta_{p}^{2}$ = 0.187). Greater values were observed for CAFF and PLAC at CMJ pre, compared to CMJ post and CMJ post-3 (*p* < 0.001; Figure 3B), with larger MP at CMJ pre for CAFF, compared to PLAC (2344.55 ± 457.9 W vs. 2237.54 ± 433.98 W; *p* = 0.006; Figure 3B). Moreover, a lower MP was detected for CAFF and PLAC were found at CMJ post, compared to CMJ post-3 (*p* < 0.001).

## DISCUSSION

This present study revealed an effect of acute CAFF supplementation on peak power (W_peak_), time to reach peak power (T_W_peak_), and mean power (W_mean_) recorded during a WT, as well as on PP and MP measured in a pre-exercise CMJ test. After the WT (L-post- and L-post-3.5), BLa concentrations were also higher in participants subjected to CAFF supplementation compared to PLAC. However, no differences between CAFF and PLAC conditions were found for muscle fatigue measured through PP and MP at the points CMJ post- and CMJ post-3 conditions. Collectively, these results suggest that CAFF increases sport performance in a high-intensity anaerobic effort (WT) without affecting the fatigue levels, which would in turn favour athletes’ functional adaptions to training.

Several studies have confirmed the ergogenic effects of CAFF when executing a WT [10, 11]. Our results indicate that CAFF (6 mg·kg^-1^) increased W_peak_ (+ 3.22%) and W_mean_ (+ 2.7%) and reduced T_W_peak_ (-18.76%) in the WT. The results are in agreement with those of a meta-analysis [11] reporting increases of 3% and 4% in W_peak_ and W_mean_ in WT performance after CAFF. When examining the kinetics of power output production during the WT, our results are in line with those of a study by San Juan et al. [26], where W_peak_ was observed in the 2^nd^ split (5–10 s) whereas the maximum mean power output was reached in the 3^rd^ split (10–15 s), preceding the power descent until the end of the WT. These data suggest that W_peak_ occurs when PCr stores are the main energy source [27]. During the second part of the WT, the critical reduction in PCr pools in the muscle promotes ADP accumulation, which is associated with an increase in the contribution of the glycolytic pathway to obtain ATP. This might be explained by a potential accumulation of metabolic end-products (i.e., H ^+^), which is reflected in the diminished power output production observed in the last splits of this study. H ^+^ ions may compete with Ca^2 +^ for the myosin-binding site, impairing the muscle contraction process, as well as inhibiting PCr resynthesis and phosphofructokinase activity, which are key parts of the glycolytic metabolism [8]. The sum of all these factors could partially explain muscle fatigue during the WT [10], and the consequent decline in performance. Considering that CAFF enhances neuromuscular recruitment [28] and increases Ca^2 +^ bioavailability in the myoplasm [29], it could be possible that the increased W_peak_ observed after CAFF supplementation is mediated by enhanced muscular function. In fact, improved performance in a time-to-exhaustion cycling test has recently been reported [30], revealing a lower electromyographic signal which suggests enhanced muscle contraction efficiency. The improvement in W_mean_ in response to CAFF might be related to greater mobilization of Ca^2 +^ in the sarcoplasmic reticulum [11], which would offset the adverse effects of H ^+^ [8] and facilitate muscle contraction. However, mechanisms that could explain caffeine’s ergogenic effects seem to take place outside the muscle cell [31]. In this sense, the central factors associated with the inhibitory mechanisms of adenosine receptors [3] that lead to reduced perceived exertion [4] seem to be related to the increased W_mean_. Since lactate production is related to higher demands of ATP production by the glycolytic and phosphagen systems [32], the higher BLa concentrations detected after CAFF compared to PLAC at L-post-0.5 and L-post-3.5 could reflect the improved performance observed for CAFF during the WT.

Regarding CMJ performance, enhanced PP and MP production after CAFF intake was observed in the CMJ pre. Our data are in agreement with those of previous studies that reported increased power production in male [33] and female [34] volleyball players, female soccer players [35], and in young [36], and recreationally trained athletes [37]. A recent study analysed the effect of CAFF on CMJ performance and the duration of jump phases, reporting increased jump height, MP, PP and maximal velocity before taking off, without changes in the duration of the different jump phases (i.e., eccentric, isometric and concentric phases) [38]. The exact mechanism responsible for the effect of caffeine ingestion on jump height performance is not known. Since PCr stores and RPE cannot affect performance at CMJ pre, we speculate that the positive effect of CAFF on intra- and inter-muscular coordination during muscle contractions may result from improved motor unit recruitment due to increased Ca^2 +^ bioavailability in the myoplasm, which could act as a synergistic mechanism leading to enhanced jump ability [38]. However, using CMJ performance as an indicator of fatigue, no differences between the CAFF and PLAC conditions were observed for MP and PP at CMJ post- and CMJ post-3, which in turn were both lower compared to CMJ pre. These results are in line with a previous study where reduced CMJ performance was detected after a WT in elite athletes, with no differences emerging between CAFF and PLAC conditions [26]. It is plausible that the accumulation of ADP and H ^+^ could affect muscular contraction function during CMJ performed after the WT. At the metabolic level, the absence of PCr after the WT could partially explain the impaired performance observed in this study at CMJ post and CMJ post-3. Similarly, considering the PCr resynthesis kinetics, the small recovery in jump ability observed at CMJ post-3 is likely the consequence of a partial resynthesis of PCr reserves [39], whereas the decreased performance compared to CMJ pre would reflect NMF [23]. The results observed for jump ability in this study and those previously reported by San Juan et al. [26] represent important findings for sports training, as the participants of the present study who ingested caffeine were able to perform the WT with higher workloads, being their levels of fatigue (measured through the CMJ) similar to those recorded in the placebo condition, and thus leading to the suggestion that CAFF may mitigate the fatigue associated with high-intensity exercise. Although caffeine interventions can help support higher physiological workloads during high-intensity training sessions [40], it is important to highlight that in our study there was no mitigating effect of CAFF on decreasing fatigue. Therefore, the use of CAFF repeatedly in high-intensity sessions should be carefully considered to avoid the risk of negative adaptations to training [41] which in the medium term could lead to symptoms of non-functional overreaching [42].

Our results indicate that acute caffeine supplementation (6 mg·kg^-1^) enhances performance and increases workload without affecting fatigue levels. Thus, CAFF could be a nutritional strategy used together with a short period of overload training (i.e., functional overreaching). In this regard, athletes could be able to train with higher workloads and enhance the supercompensation effects after an adequate recovery period. Nevertheless, this study has some limitations. The effect of CAFF on Ca^2 +^ bioavailability has been observed in vitro and so it is necessary to confirm this effect in humans during exercise. In this regard, different studies have reported that CAFF may affect several psychophysiological mood variables and psychological responses, including a modification of the relationship between workload and RPE during high-intensity anaerobic efforts [43]. Therefore, future studies should assess the potential influence of the psychological responses to CAFF supplementation on the post-exercise fatigue levels.

## CONCLUSIONS

Acute supplementation with 6 mg·kg^-1^ of caffeine improved performance in a Wingate test (i.e., W_peak_, T_W_peak_ and W_mean_), and increased pre-exercise jump ability (CMJ) compared to placebo, with no effect on post-exercise muscular fatigue. Hence, CAFF could increase workload without affecting fatigue levels. Thus, using CAFF, athletes might be able to train with higher workloads and enhance the supercompensation effects after an adequate recovery period.

## Conflict of Interest Declaration

We declare that there are no conflicts of interest relevant to the content of this article.
